# Supplementary material for: The NAMPT Inhibitor FK866 in Combination with Cisplatin Reduces Cholangiocarcinoma Cells Growth
Source: Cells. 2023 Feb 28;12(5):775. doi: 10.3390/cells12050775 (PMC10001024; doi:10.3390/cells12050775)
Supplement: Supplementary file 1 [file cells-12-00775-s001.zip › cells-2098350-supplementary.pdf]

Patient Characteristics and NAMPT scoring

Supp. Table S1

	TMA Score <= 2.5 (N=44)	TMA Score > 2.5 (N=66)	Total (N=110)	p value
Age				0.500
N	44	66	110	
Mean (SD)	61.0 (11.7)	62.7 (11.8)	62.0 (11.8)	
Median	62.5	64.0	63.5	
Range	35.0 - 78.0	28.0 - 86.0	28.0 - 86.0	
Gender				0.243
Female	19 (43.2%)	36 (54.5%)	55 (50.0%)	
Male	25 (56.8%)	30 (45.5%)	55 (50.0%)	
Hepatitis..B				0.766
Missing	34	43	77	
9	0 (0.0%)	1 (4.3%)	1 (3.0%)	
No	9 (90.0%)	19 (82.6%)	28 (84.8%)	
Yes	1 (10.0%)	3 (13.0%)	4 (12.1%)	
Hepatitis..C				0.798
Missing	33	40	73	
9	0 (0.0%)	1 (3.8%)	1 (2.7%)	
No	9 (81.8%)	20 (76.9%)	29 (78.4%)	
Yes	2 (18.2%)	5 (19.2%)	7 (18.9%)	
PSC				0.283
Missing	33	45	78	
No	7 (63.6%)	17 (81.0%)	24 (75.0%)	

Yes	4 (36.4%)	4 (19.0%)	8 (25.0%)	
Cirrhosis				0.692
Missing	33	43	76	
No	9 (81.8%)	20 (87.0%)	29 (85.3%)	
Yes	2 (18.2%)	3 (13.0%)	5 (14.7%)	
CCA..Classification				0.036
Intrahepatic	44 (100.0%)	66 (100.0%)	110 (100.0%)	
TNM.Stage...documented.				0.087
Missing	7	11	18	
STAGE I	14 (37.8%)	20 (36.4%)	34 (37.0%)	
STAGE II	8 (21.6%)	12 (21.8%)	20 (21.7%)	
STAGE III	3 (8.1%)	4 (7.3%)	7 (7.6%)	
STAGE IVA	4 (10.8%)	16 (29.1%)	20 (21.7%)	
STAGE IVB	6 (16.2%)	1 (1.8%)	7 (7.6%)	
STAGE UNKNOWN	2 (5.4%)	2 (3.6%)	4 (4.3%)	
T.Stage				0.315
Missing	4	7	11	
T1	17 (42.5%)	37 (62.7%)	54 (54.5%)	
T2	0 (0.0%)	1 (1.7%)	1 (1.0%)	
T2a	4 (10.0%)	3 (5.1%)	7 (7.1%)	
T2b	12 (30.0%)	12 (20.3%)	24 (24.2%)	
T2B	1 (2.5%)	0 (0.0%)	1 (1.0%)	
T3	5 (12.5%)	5 (8.5%)	10 (10.1%)	
TIII	1 (2.5%)	0 (0.0%)	1 (1.0%)	
TX	0 (0.0%)	1 (1.7%)	1 (1.0%)	
N.Stage				0.325

Missing	4	7	11	
N0	29 (72.5%)	43 (72.9%)	72 (72.7%)	
N1	6 (15.0%)	14 (23.7%)	20 (20.2%)	
No	1 (2.5%)	0 (0.0%)	1 (1.0%)	
NX	1 (2.5%)	0 (0.0%)	1 (1.0%)	
Unknown	3 (7.5%)	2 (3.4%)	5 (5.1%)	
M.Stage				0.011
Missing	4	7	11	
M0	34 (85.0%)	58 (98.3%)	92 (92.9%)	
M1	6 (15.0%)	1 (1.7%)	7 (7.1%)	
Surgical.Margins				0.960
Missing	16	30	46	
Involved by invasive carcinoma	3 (10.7%)	4 (11.1%)	7 (10.9%)	
Uninvolved by invasive carcinoma	25 (89.3%)	32 (88.9%)	57 (89.1%)	
CEA				0.613
Missing	24	33	57	
N	20	33	53	
Mean (SD)	2.2 (2.0)	13.5 (52.9)	9.3 (41.9)	
Median	1.5	1.6	1.6	
Range	0.5 - 7.9	0.5 - 303.4	0.5 - 303.4	
CA19_9				0.734
Missing	8	14	22	
N	36	52	88	
Mean (SD)	612.6 (2275.4)	4882.7 (30675.8)	3135.8 (23625.5)	

Median	62.5	54.5	57.5	
Range	1.0 - 13514.0	1.0 - 221600.0	1.0 - 221600.0	
Tumor_size_cm_				0.645
N	44	66	110	
Mean (SD)	7.2 (3.5)	7.0 (3.9)	7.1 (3.7)	
Median	6.9	5.8	6.4	
Range	2.0 - 20.0	0.5 - 20.0	0.5 - 20.0	
Tumor_Grade				0.953
Missing	0	1	1	
N	44	65	109	
Mean (SD)	2.9 (0.5)	2.9 (0.5)	2.9 (0.5)	
Median	3.0	3.0	3.0	
Range	2.0 - 4.0	1.0 - 4.0	1.0 - 4.0	
Vascular_invasion				0.713
Missing	2	0	2	
N	42	66	108	
Mean (SD)	0.2 (0.7)	0.2 (0.6)	0.2 (0.6)	
Median	0.0	0.0	0.0	
Range	0.0 - 2.0	0.0 - 2.0	0.0 - 2.0	
AFP				0.583
Missing	16	18	34	
N	28	48	76	
Mean (SD)	3.1 (1.9)	3.9 (6.6)	3.6 (5.3)	
Median	3.0	2.6	2.7	
Range	0.2 - 8.4	0.2 - 45.6	0.2 - 45.6	
TB				0.298

Missing	2	3	5	
N	42	63	105	
Mean (SD)	1.1 (2.3)	0.8 (1.2)	0.9 (1.7)	
Median	0.6	0.5	0.6	
Range	0.1 - 14.8	0.1 - 9.1	0.1 - 14.8	
ALB				0.607
Missing	11	8	19	
N	33	58	91	
Mean (SD)	3.6 (1.5)	4.1 (0.8)	3.9 (1.1)	
Median	4.2	4.3	4.3	
Range	0.4 - 4.7	0.3 - 4.9	0.3 - 4.9	
ALT				0.380
Missing	14	20	34	
N	30	46	76	
Mean (SD)	4.5 (3.9)	5.4 (7.3)	5.1 (6.2)	
Median	3.1	3.7	3.6	
Range	0.6 - 17.7	1.4 - 50.7	0.6 - 50.7	
PT				0.448
Missing	4	2	6	
N	40	64	104	
Mean (SD)	10.1 (6.6)	9.3 (3.6)	9.6 (4.9)	
Median	9.6	9.6	9.6	
Range	0.9 - 46.3	0.1 - 24.5	0.1 - 46.3	

TMA score by TNM stage (either as continuous or dichotomized by median values)

	Stage I-II (N=76)	Stage III-IV (N=35)	Total (N=111)	p value
tma.score				0.941
Missing	0	1	1	
N	76	34	110	
Mean (SD)	2.6 (0.6)	2.6 (0.5)	2.6 (0.5)	
Median	3.0	3.0	3.0	
Range	1.0 - 3.0	1.5 - 3.0	1.0 - 3.0	

	TMA Score <= 2.5 (N=44)	TMA Score > 2.5 (N=66)	Total (N=110)	p value
stage_high				0.801
Stage I-II	31 (70.5%)	45 (68.2%)	76 (69.1%)	
Stage III-IV	13 (29.5%)	21 (31.8%)	34 (30.9%)	

Supp. Figure S1

Overall Survival by TMA Score

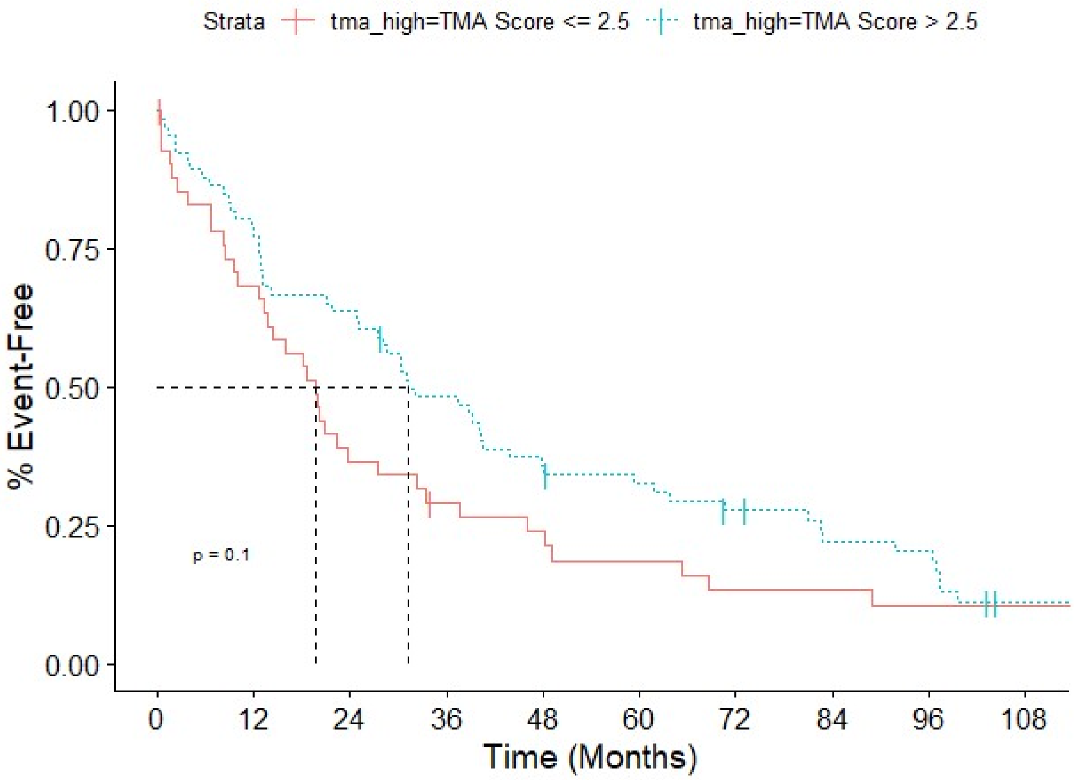

Supp. Figure S2

Recurrence-free Survival by TMA Score

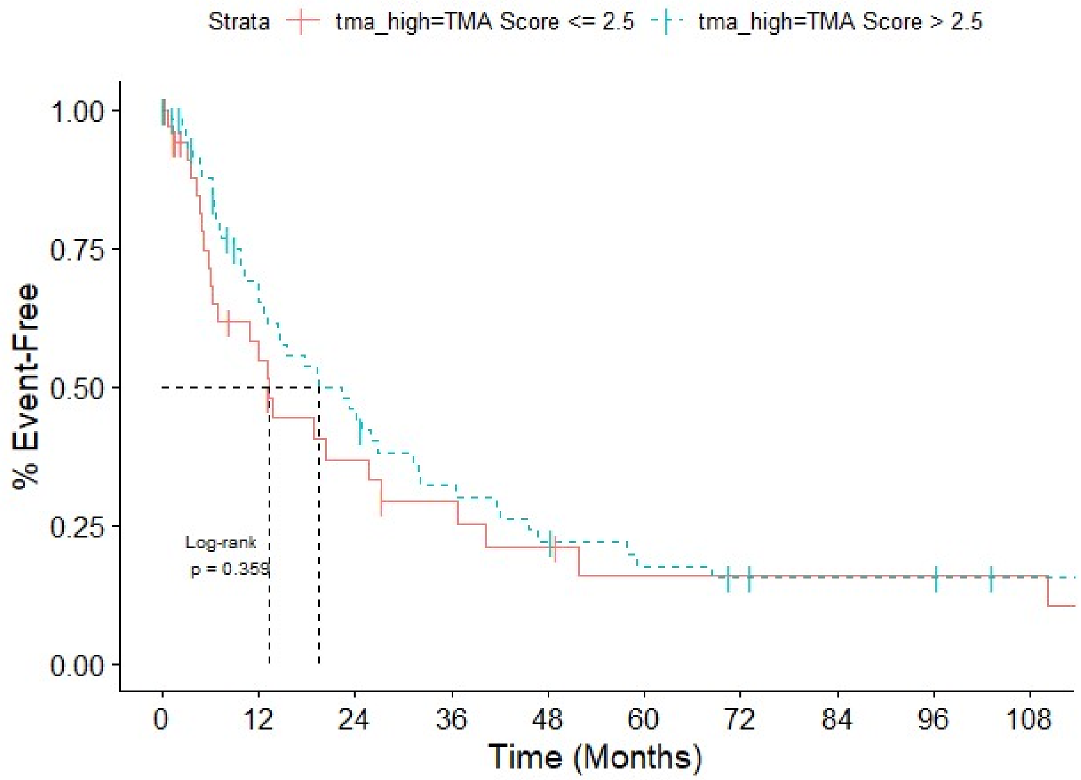

Supp. Figure S3

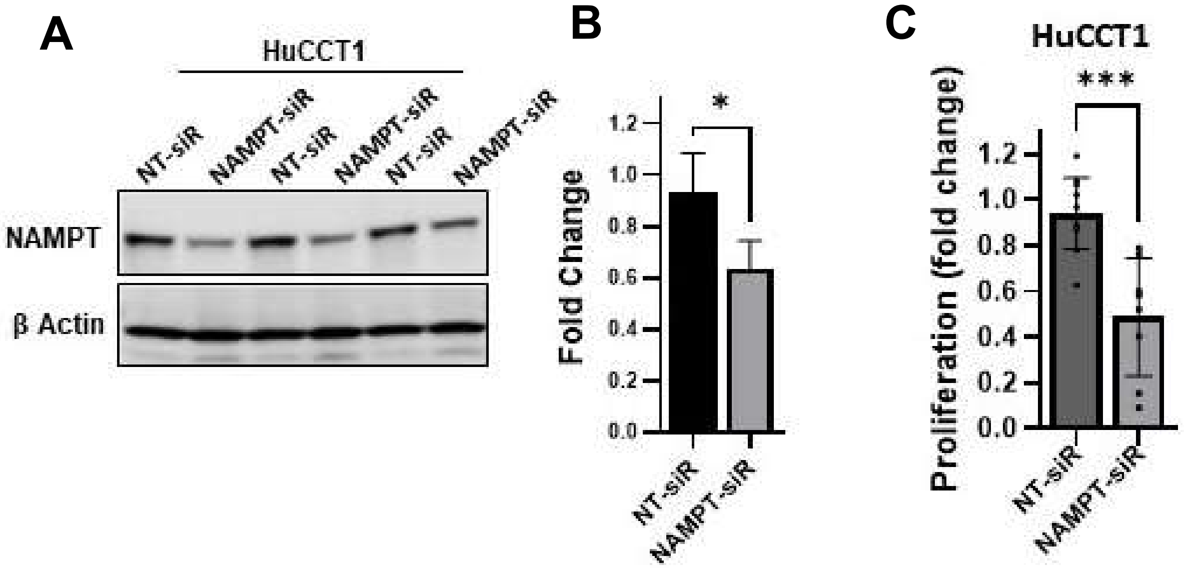

A, NAMPT expression was assessed by western blotting in, HuCCT1 cells transfected with NAMPT-siRNA or NT-siRNA. **B,** densitometry analysis. **C,** HuCCT1 cells were transfected with NAMPT-siRNA or NT-siRNA, and cell growth was assessed after 72 hours using MTS assay. (* $p< 0.05$, *** $p<0.001$)

Supp. Figure S4

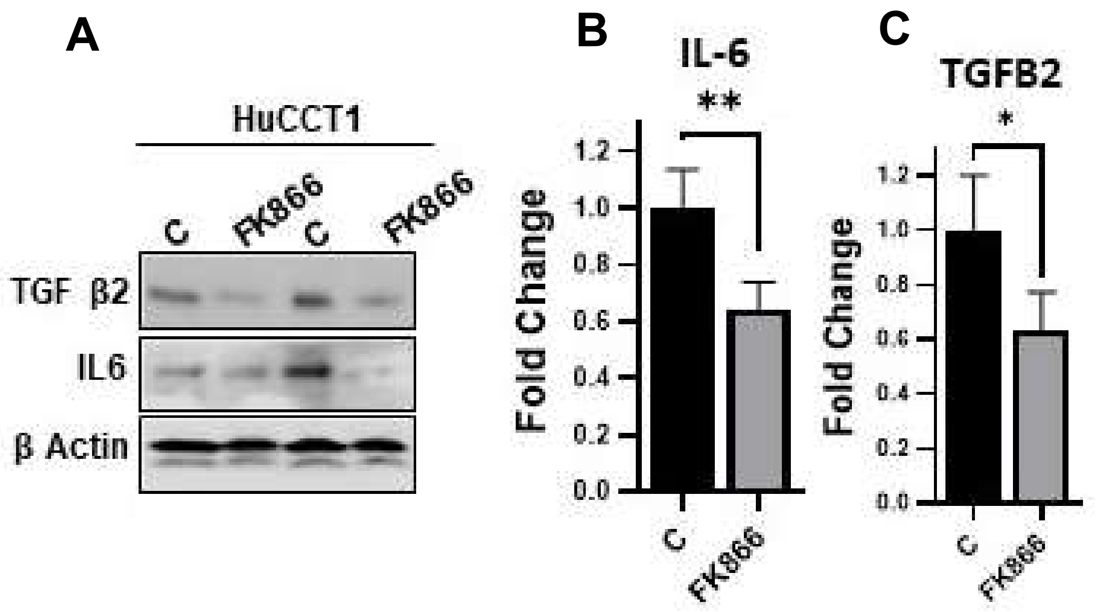

A, B & C. TGF b2 and IL6 expression were measured by western blotting in HuCCT1 cells treated with FK866 or vehicle control and densitometry analysys performed. (* $p < 0.05$, ** $p < 0.01$)
